# Identifying species from the air: UAVs and the very high resolution challenge for plant conservation

**DOI:** 10.1371/journal.pone.0188714

**Published:** 2017-11-27

**Authors:** Susana Baena, Justin Moat, Oliver Whaley, Doreen S. Boyd

**Affiliations:** 1 Royal Botanic Gardens, Kew, Richmond, Surrey, United Kingdom; 2 School of Geography, University of Nottingham, Nottingham, United Kingdom; Universita degli Studi di Trento, ITALY

## Abstract

The Pacific Equatorial dry forest of Northern Peru is recognised for its unique endemic biodiversity. Although highly threatened the forest provides livelihoods and ecosystem services to local communities. As agro-industrial expansion and climatic variation transform the region, close ecosystem monitoring is essential for viable adaptation strategies. UAVs offer an affordable alternative to satellites in obtaining both colour and near infrared imagery to meet the specific requirements of spatial and temporal resolution of a monitoring system. Combining this with their capacity to produce three dimensional models of the environment provides an invaluable tool for species level monitoring. Here we demonstrate that object-based image analysis of very high resolution UAV images can identify and quantify keystone tree species and their health across wide heterogeneous landscapes. The analysis exposes the state of the vegetation and serves as a baseline for monitoring and adaptive implementation of community based conservation and restoration in the area.

## 1. Introduction

The Pacific Equatorial dry forest of Northern Peru (and South Western Ecuador) is recognised as a unique dry forest ecosystem, rich in endemic flora and biodiversity [1]. The region is a vital and unique ecosystem within the Latin American biome of seasonally dry tropical forest and that is one of the most threatened, and yet understudied and very poorly surveyed, tropical forests worldwide with less than 10% of the original extent remaining regionally [2]. The Equatorial dry forest of Peru also provides a wide range of livelihoods and ecosystem services to local communities [3]. However, agro-industrial expansion, over-exploitation of natural resources (for firewood and agriculture land) and climatic extremes (e.g., ENSO events) has left forest relicts and a mosaic of associated arid land vegetation which are now highly threatened and vulnerable [4]. Furthermore, over the last ten years the forest has been experiencing die-back of its keystone species: the Algarrobo (*Prosopis pallida*) upon which communities and livelihoods have historically depended [5]. This is likely due to the combined impact of drought, associated climate change and defoliating plagues, especially *Enallodiplosis discordis* (*Diptera*: *Cecidomyiidae*). The Algarrobo has been the lynch-pin of rural livelihoods as a multipurpose tree [6] providing an annual rich harvest of nutritious pods that are used for food and especially for animal forage, as well as a foodstuff for human consumption. Of particular note is that the Algarrobo pods are an essential forage component that allows the ranching of animals on the very poor soils with limited water that prevails in this area. Conversely, other species such as Sapote (*Colicodendron scabridum*) and Overo (*Cordia lutea*) appear to be thriving, possibly due to atmospheric CO_2_ enrichment and increasing temperatures [7], especially during summer rains with the free nitrogen released from *Algarrobo* die-back [7].

A highly complex and dynamic ecosystem now exists that requires close monitoring to quantify change and respond effectively with adaptation strategies in order to manage the decline in Algarrobo-dependent biodiversity and sustain community livelihoods. In this context a monitoring system needs to be capable of the following: (i) identifying individual tree species across the landscape; (ii) mapping species association and plant community whilst assessing plant health and (iii) quantifying population dynamics in response to ongoing land management and climate change. Such monitoring is essential from a scientific perspective (e.g.to target and design restoration and reforestation efforts to preserve and restore the native forest relict), but must also be able to communicate to stakeholders the best actions (such as changes in seed banking, reforesting options, irrigation and livestock management) to maintain resilience of ecosystem services. Specifically, the monitoring system must provide spatial data on the principal keystone species, *Algarrobo* (including on their current mortality rates), as well as on *Sapote* other dominant species and do so in a timely fashion.

This detailed ecosystem assessment has traditionally been carried out through field survey, but collection of field data is costly in time, labour and resources [8] and so remote sensing has been much advocated as an approach, as it can characterise an ecosystem in an efficient, systematic, repeatable and spatially exhaustive manner [9]. Remote sensing based ecosystem assessments can be conducted at a range of spatial scales, from global [10–13] to more localised studies [14–19]. However, discriminating between species and quantifying individual plants using remote sensing requires specific acquisition parameters (e.g., data at a sufficient resolution) and processing approaches. Very high spectral resolution (hyperspectral) data have been successful in the identification of canopy tree species [20–23]. Alternatively, very high spatial resolution data is perhaps more accessible and has the potential to be a viable tool for species determination [24–26]. Further, since species identification can be greatly improved by employing the three dimensional information of the vegetation, technologies affording three dimensional measurements (e.g., light detection and ranging (LiDAR) technology) have been widely used [27–30]. However, in all these cases, the costs and availability of the technology would be financially and structurally prohibitive for most conservation efforts [31], yet the potential of remote sensing to support conservation effort has been recognised [32] (Rose et al., 2014). In this paper, we investigate the effectiveness of remote sensing from unmanned aerial vehicles (UAVs) which have the potential to provide accurate and fast analysis and monitoring of ecological features at a cost [33] that can be accessible to most conservation applications and researchers in developing countries [34].

The use of UAVs as a remote sensing approach is now gaining traction. These type of systems are especially well suited to and most commonly used for precision agriculture where the technology is developing fast [35,36] but they have also been employed in different ecological [33], environmental [37] and conservation [34] applications where UAV systems have been identified to have the potential to revolutionize these fields [33]. Low altitude flights can easily provide the sub-meter spatial resolution that allows individual plants to be identified [38]. Also, different sensing payloads can deliver multispectral images, ranging from the visible (RBG) band, to the near infrared (NIR) through to the thermal infrared and microwave [39] although often, because of the low-cost requirements and limited weight capabilities of these systems, modified consumer digital cameras are used [40], with enhancement to their spectral resolution by using or removing filters to provide RBG and NIR bands [41]. Three dimensional information can be derived from overlapping images taken with uncalibrated consumer-grade cameras using newly developed algorithms [42], in particular structure from motion [43,44], providing extra valuable information on vegetation structure and condition. Moreover, the system can be deployed on demand, providing temporal flexibility to respond to specific needs such as seasonal or climatic circumstances (e.g., an El Niño event) or to monitor interventions (e.g. tree planting or cattle exclusion) [45].

Despite the promise offered by using UAVs, obtaining meaningful ecological information from the captured data remains challenging and so far has predominantly relied on visual interpretation of the imagery acquired, particularly when it comes to quantifying number of individuals [33]. This can be very time consuming and become unfeasible in larger areas with large number of individuals. Further, traditional pixel-based image analysis techniques have limitations when processing such high resolution datasets [46,47] where image targets (such as individual trees) are larger than the pixel size. Combining pixels into groups to form objects could be a way forward to analysing this type of data. Additional spectral information contained in an object (e.g. mean spectral values, variance, mean ratios…) can be taken into account along with added spatial and contextual information for the objects (distance, shapes, size, texture, etc.). The inclusion of this extra information is critical when uncalibrated consumer-grade digital cameras are used as these imaging sensors acquire images that have high spatial resolution but lack radiometric quality [48]. Thus object-based image analysis (OBIA) techniques have demonstrated great potential to automatically extract information from very high resolution images [49,50], including those captured by UAVs [51,52].

In this paper we present an efficient and affordable approach to identify and quantify individual trees across the dry forest, as well as mortality rates of its keystone species. We seek to add weight to the growing evidence on the real potential of UAV’s for plant conservation, with the view that this could form the foundation of a monitoring system across this particular landscape.

## 2. Materials and methods

### 2.1. Study area

**Fig 1:**
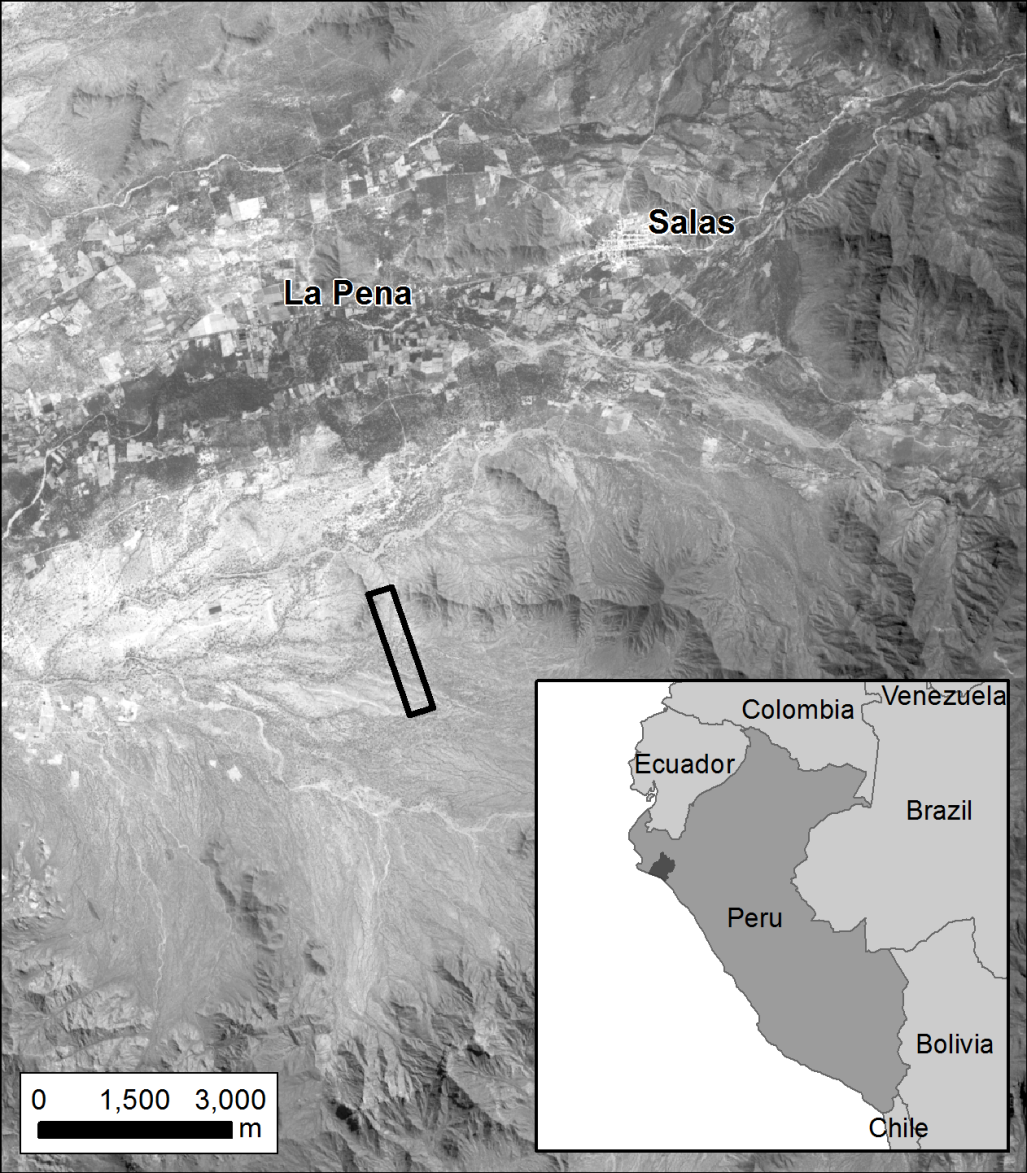
Study site and country map: Country map shows the Peruvian region of Lambeyeque. Main map for context, UAV flight is highlighted in black, overlaid with a Landsat Panchromatic image.

The community forest (comunidad campesina) of San Francisco de Asis (CCSFA) in the Lambayeque region in Northern Peru was the focus of investigation (Fig 1). Full permission to carry out UAV flights and botanical collections in the area as part of this study was granted by the president of San Francisco de Asis Community forest. The CCFA is delimited by a low mountain range and it is one of a series of arid slopes and a dry lower watershed basin on the western side of the Andes. Rainfall is very low and rarely exceeds 400 mm (except during some El Niño events), generally falling between December to April. UAV data were captured for an 80 ha area (centred on 79° 38’W, 6° 20’ S, datum WGS84) within the proposed CCSFA community reserve.

**Fig 2:**
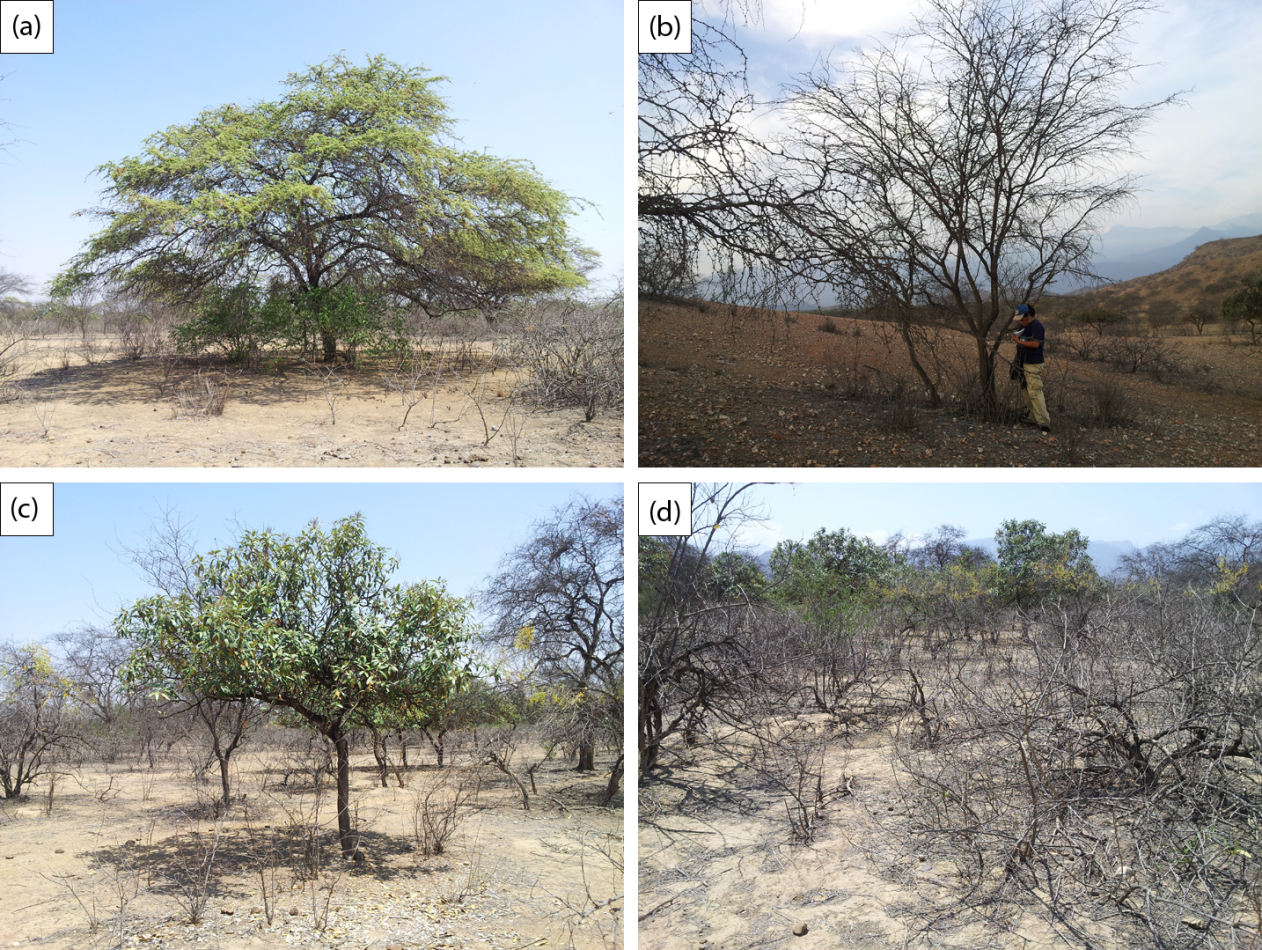
Target species: (a) Alive Algarrobo (b) Dead Algarrobo (c) Sapote (d) Overo.

The arboreal vegetation associated with the study area is dominated principally by *Prosopis pallida* (Algarrobo) with *Colicodendron scabridum* (Sapote), and *Cordia lutea* (Overo) as a shrub which occurs ubiquitously along the network of ephemeral run-off streams that transverse the bajada (Fig 2). *Vallesia glabra* (Cun cun) occurs as a *Prosopis pallida* sub canopy shrub, and amongst this vegetation, at low density occur *Cynophalla flexuosa* (Sune)—2–5 individuals per ha, *Grabowskia boerhaaviifolia* (Canutillo or Palo negro) occasionally and *Parkinsonia praecox* (Palo verde)—4–10 individuals per ha. At very low density on the lower plain at less than one tree per 5 ha are *Loxopterigium huasango* (Hualtaco) and *Bursera graveolens* (Palo santo), and relict large columnar cactus *Armatocereus* aff. *cartwrightianus* and *Neoraimondia arequipensis*).

**Fig 3:**
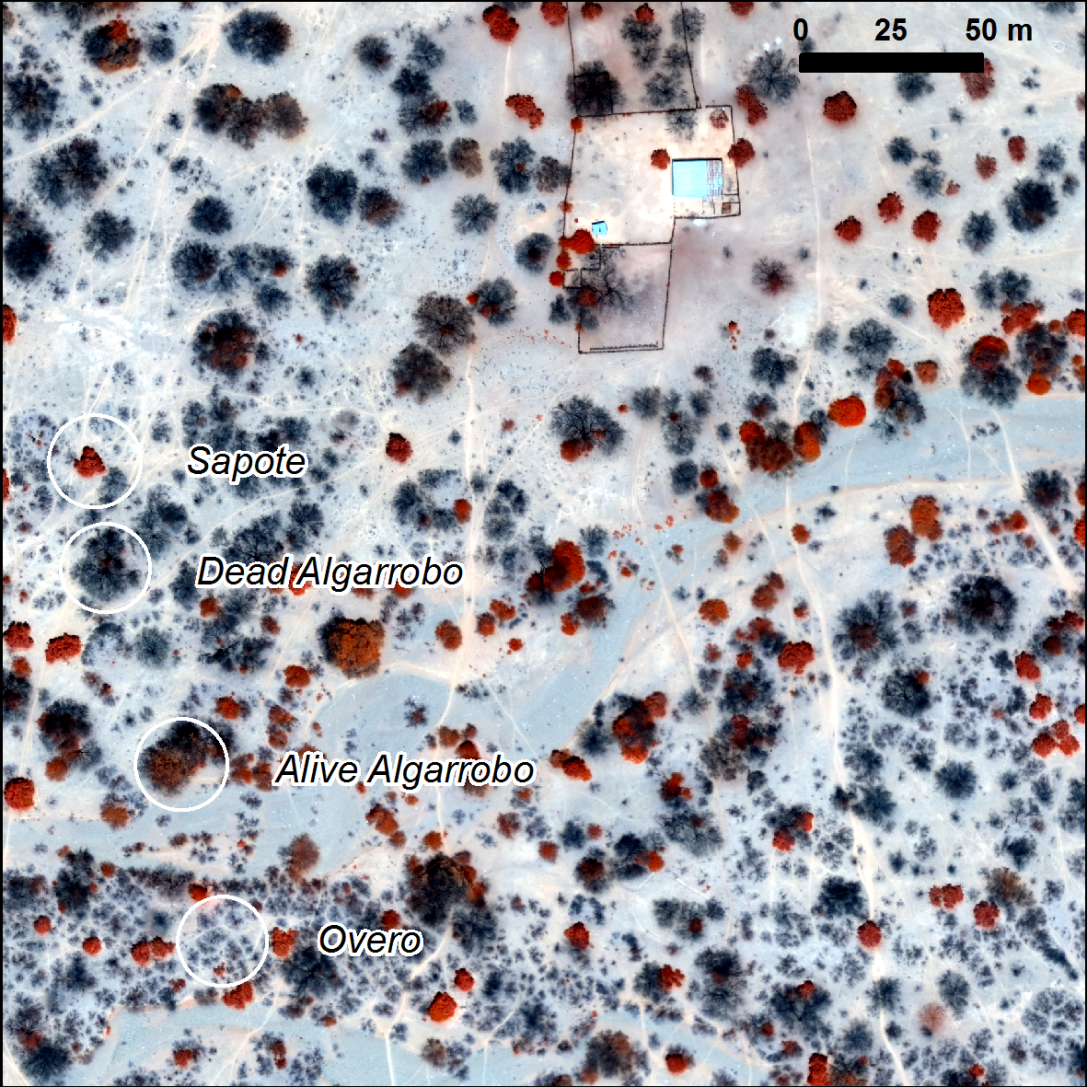
Target species from the UAV image.

In this study and for the purpose of identification from UAV images the focus will be on the dominant species. Each of these plant species have a morphology that could help in any identification using remotely sensed data, such as that captured by an UAV. As such, there are several important plant features that can be used for identification of each of the species of interest (Fig 3):

**Algarrobo** *(Prosopis pallida)*.: This is a non-deciduous tree of up to 15 meters high with a very distinctive star shaped crown when viewed from above. This distinctive shape is modified as a result of the dieback affecting this keystone species.

***Overo*** *(Cordia lutea*) that could get misclassified as *Prosopis pallida* when observed from above but can easily be distinguished as it is deciduous and only up to 2 meter high. It grows along runnels forming a distinctive reticulated pattern

**Sapote** *(Capparis scrabrida)*: This evergreen tree is very distinctive with compact and round or sub oval shaped crown with large sclerophyllous leaves. It tends to grow partitioned and isolated, this feature is exaggerated in this site through the use of the dense shade for cattle sheltering the extreme midday heats.

### 2.2. Datasets: Remote sensing with UAV and supporting ground data

The UAV deployed was the fixed-wing eBee system (https://www.sensefly.com/). This autonomous system is operated by a propeller activated by an internal electric motor. It requires hand launch for take-off and landing is either linear or circular in a relatively clear area (i.e. bare ground or grass). Its efficient aerodynamics allows for long flight durations and high speeds. It can cover up to 12 Km^2^ in a single flight and by altering the flying altitude can be used to acquire very high resolution images (i.e. up to 1.5 cm). The imaging sensor used on board was a Canon S110 RE, a customised very light automatic consumer-grade digital camera providing blue, green and red-edge band data. The red-edge band data is obtained by adding an optical red-light-blocking filter in front of the sensor, resulting in red-edge, green and blue images [41]. It has a resolution of 12 MP with a ground resolution at 100m of 3.5 cm/px. The sensor size is 7.44 x 5.58 mm with a pixel pitch of 1.86 um. The UAV was deployed during the dry season (November) to ensure maximum discrimination between deciduous and evergreen vegetation. The flight mission was planned using eMotions software over images imported from Google Earth (Google Earth, 2011) and SRTM DEM [53]. Flight altitude was set up to 260m to provide a ground resolution of approximately 8 cm over the study area and images acquired at 75% forward overlap and 80% side overlap. The ground resolution of 8 cm was chosen to provide ultra-high resolution (both in the resultant point cloud and mosaics), but also to cover a large enough area to include established control plots and demonstrate the benefits of UAV monitoring. The high level of overlap allows 3D reconstruction of point clouds to a higher accuracy, allowing objects in each image to be observed in at least 5 other images and thus affording structure from motion analysis.

**Fig 4:**
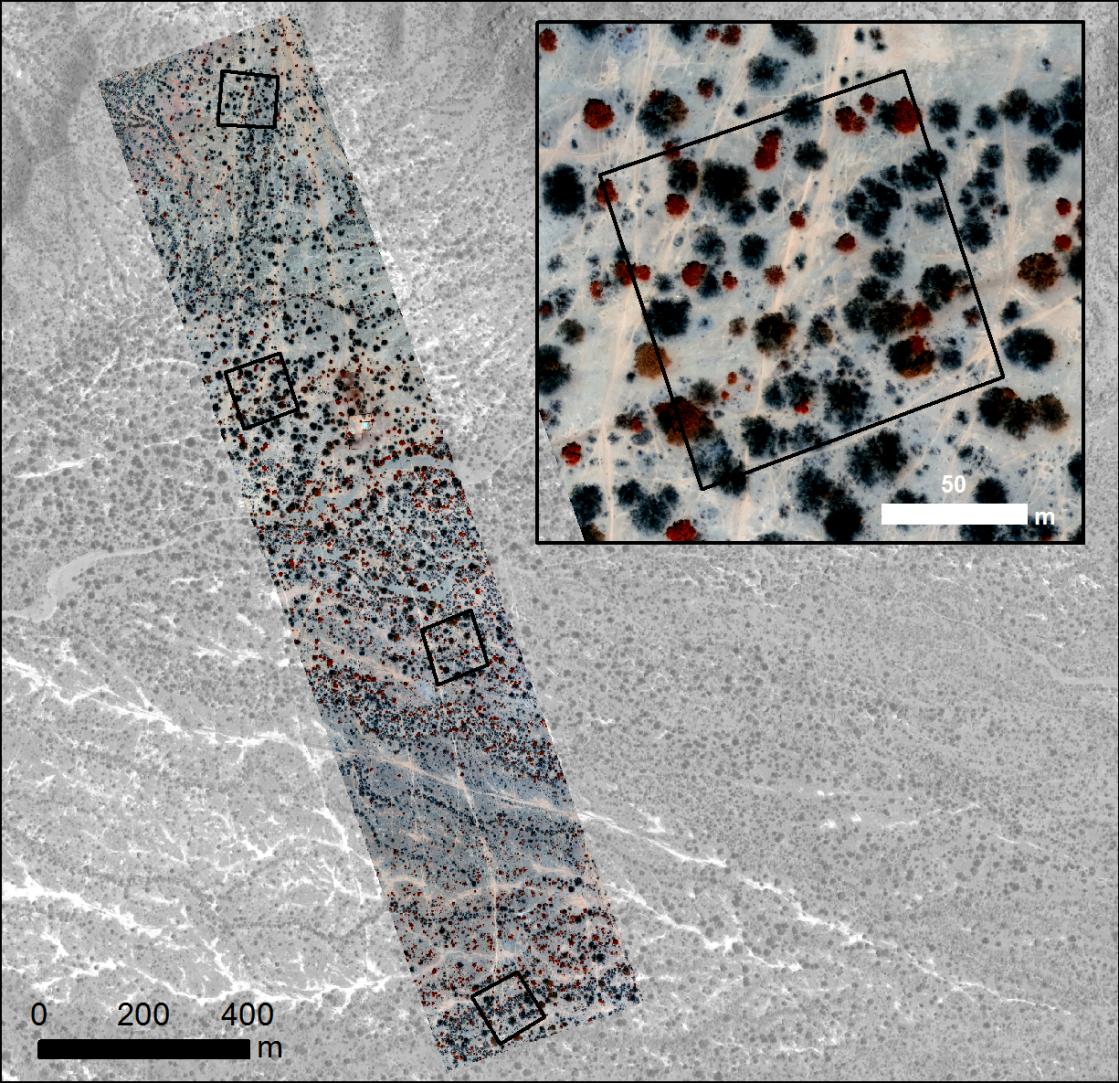
UAV flight (red edge) over the study area and plot distribution (from top to bottom: Plots 1, 3, 4, 2). Background image: panchromatic World View image.

Temporally coincident to the UAV flights, four experimental plots were measured. These 1 Ha (100m x 100m) plots were evenly distributed spatially across the study site (Fig 4) recording the presence and species of every mature tree with (> 25 cm girth at 50 cm high) along with the average crown spread. For every *Algarrobo* tree the “degree of health” was also recorded on a 1 to 3 scale (1: healthy tree, 2: infected tree, 3: dead tree). For the shrub layer, presence of *Overo* was recorded where it covered an area of more than 4 square meters (2 x 2 m). In addition, a discontinuous transect throughout the length of the flight (2 Km length by 0.4 Km wide) was surveyed, recording the species of every tree found.

### 2.3. Pre-processing of remotely sensed data

The raw UAV- derived remotely sensed data were processed using eMotion's Flight Data Manager and Postflight terra (Pix4D) software to produce a geo-referenced orthorectified 3 waveband image mosaic at 8.3 cm spatial resolution (UTM 17S WGS84) and two 3D-derived elevation layers: a digital elevation model (DEM), a representation of the elevation of the ground surface or bare-earth, and digital surface model (DSM) which includes the elevation of both natural and built features. Both layers are delivered as grids with a resolution matching that of the orthorectified mosaic. Both were then used to derive a canopy height model (CHM) of the study area.

### 2.4. Species mapping from UAV data

**Fig 5:**
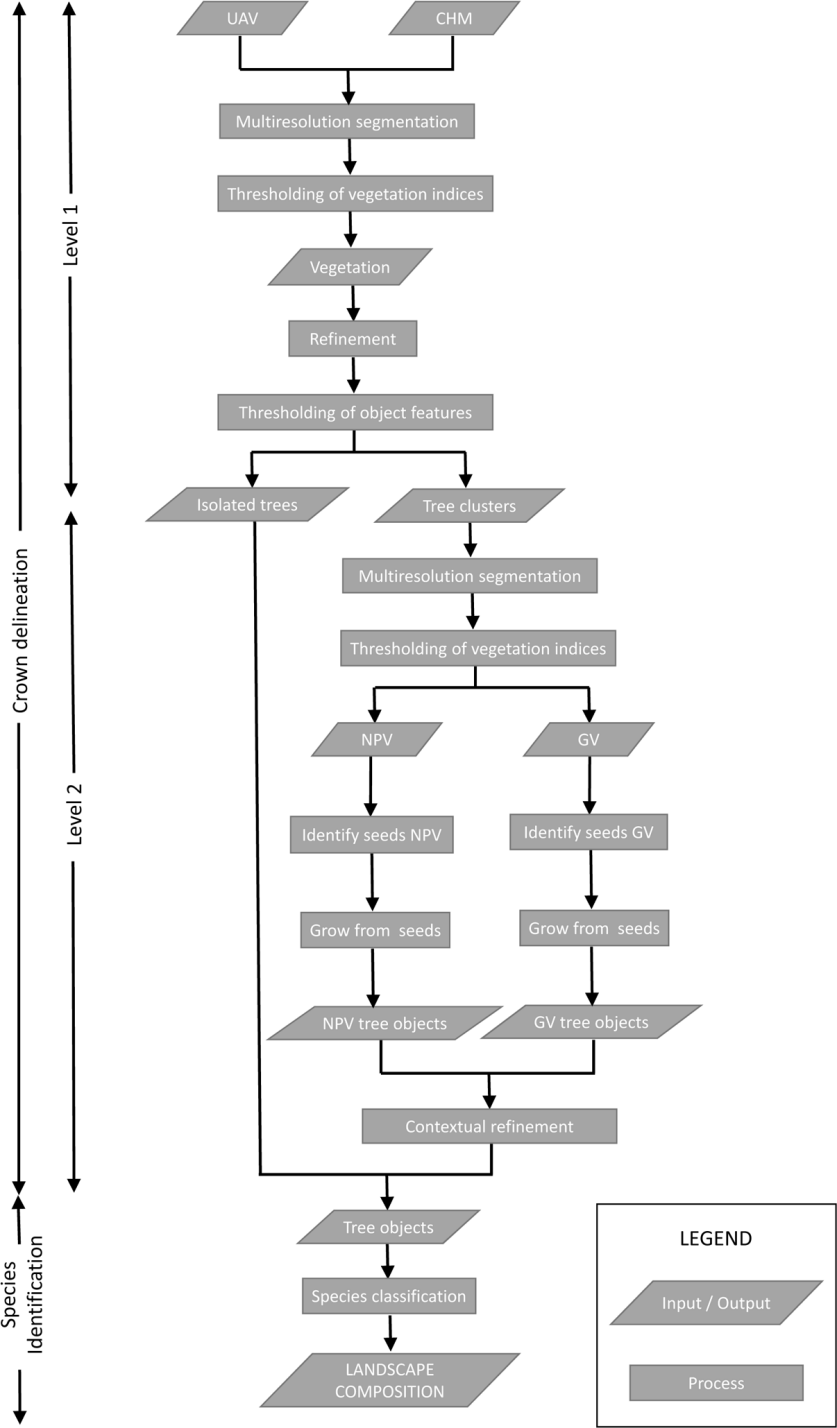
Methodology work flow.

Mapping of the target species (Algarrobo, Sapote and Overo), as well as mortality rates of the keystone Algarrobo was undertaken in two steps. The first was to identify individual trees by delineating crowns and the second was to use the individual tree crown as the basic unit for species identification [54]. Object-based image analysis provided the tools to do just this by allowing the isolation of individual homogeneous objects in the image corresponding to one particular tree crown [47]. Unlike most studies in which crowns are delineated in one single segmentation process, crown delineation here is achieved by the iteration of segmentation and classification of subsequent segments working at two different levels. A first level aims to isolate individual trees from tree clusters across the landscape using contextual information. At a more detailed second level, individual crowns are identified within these tree clusters using two different approaches according to vegetation types. This allowed for a more efficient methodology focusing processing efforts only where it is most needed, as well as using the best suited methodology for each specific vegetation type. Fig 5 and Table 1 include the methodology workflow and main processing parameters.

**Table 1:** Summary of processing parameters.

Level	Operation	Algorithm/Object metrics	Parameters	Value
L 1	Multiresolution segmentation		Layer weights	1 (B) 1 (G) 1 (RE) 1 (CHM)
			Shape	
			scale parameter	
			Compactness	
	Thresholding of vegetation indices		NPV	>0.053
			GDVI	>0.03
	Refinement	Merge Region	Vegetation	
		Remove objects		
		Merge Region		
		Find enclose by class		
		Merge Region		
	Thresholding of Object Features	Roundness	<0.9	
		Area	<30,000 pixels	
L 2	Identify seeds (NPV)	Find local extrema	Search range	20
			Extrema type	Maximum
			Feature	Mean DSM
	Identify seeds (GV)	Find local extrema	Search range	50
			Extrema type	Maximum
			Feature	NPV
	Grow from seeds	Grow region		
	Contextual Refinement	Relative border to		
		Find enclose by class		
		Remove objects		
	Species. Classification (Sapote)	Assign class	GDVI	>_0.08
	Species. Classification (Alive Algarrobo)	Assign class	GDVI	< 0.08
	Species. Classification (Dead Algarrobo)	Assign class	Area	>_10,000 pixels
			NPV	> 0.053
			CHM	>0.4
	Species. Classification (Overo)	Assign class	Area	< 10,000 pixels
			NPV	> 0.053
			CHM	< 0.4

#### Crown delineation

Level 1 (vegetation or contextual level): Firstly a multiresolution segmentation algorithm (using eCognition software) was used to create the basic image objects at a scale that allows for objects to be relatively homogeneous whilst capturing the full scene variability. These “object candidates” [55] will be transformed by further processing into meaningful objects (individual tree crowns). Vegetation is then discriminated from the background by thresholding available band ratios (from Blue, Green and Red Edge bands). Green vegetation is best discriminated using a band ratio, deviation from the normalised vegetation index (1) whiles the non-photosynthetic vegetation (deciduous and dead vegetation) using a combination of the blue and green bands (2).

1. GV (Green Vegetation) = (RE–Green) / (RE + Green)
2. NPV (Non-Photosynthetic vegetation) = (Blue–Green) / (Blue + Green)

Objects classified as vegetation were then combined and reclassified as either isolated trees (individual tree crowns) or tree clusters according to contextual object features (extent and shape). Vegetation objects smaller than the minimum mapping unit (4m^2^) are not considered for further analysis. Tree clusters were then need to be further analysed (level 2, see below) to extract individual trees within them.

Level 2 (de-clustering): Most tree crown delineation studies are built around the identification of treetops, assuming that they are at a maxima (most often using CHMs) and that they correspond to the geometric centre of the tree crown [56]. This is normally the case in coniferous trees and can be extended to evergreen vegetation but deciduous tree crowns (and this is also the case for defoliated evergreen vegetation) are relatively flat and with multiple branches that can be identified as individual trees [54]. This, added to the challenges presented by drone derived elevation data in deciduous trees prevents the use of CHM local maxima to identify hardwood tree tops. In this case, tree centres are identified using NPV local maxima. Green vegetation was very clearly identified in the elevation model therefore a more traditional CHM local maxima was used to identify tree tops.

Once tree tops have been identified, a region growing algorithm was used to delineate the respective tree crowns [57]. A further step using contextual information was then needed to refine certain tree crowns, such as infested Algarrobo which were in certain cases identified as two different trees (dead branches adjacent to branches with different levels of defoliation). The feature “relative border to” (measuring the relative border length that an object shares with neighbour objects) was particularly useful for this purpose.

#### Species identification

Once the crowns have been delineated, individual trees have additional spectral information as opposed to single pixels (eg. mean values, maximum and minimum, variance…) along with contextual information and specific object features that were to be used for species discrimination. In our case the most useful features for species discrimination was; mean values of band ratios, mean, maximum and standard deviation of CHM, shape and size, as well as contextual information such as “relative border to”. Training information for classification used data collected in a transect along the flight (based on visual interpretation), whilst further plot data was used for validation. The final classification scheme aimed to discriminate amongst four classes: Sapote, Overo, Alive Algarrobo and Dead Algarrobo.

In terms of accuracy assessment of the final species map there were two main objectives. The first was to determine the accuracy in the estimates of number of trees mapped (tree detection) and the second was to determine the level of confidence for the identification of individual species (species classification). The assessment was implemented at a plot level, as a non-site specific measurement, avoiding the need to match individual tree location [58]. Data recorded in the four plots taken at the time of the flight were used as reference. Only trees with an average crown spread greater than 4m^2^ were considered (matching the minimum mapping unit). Algarrobo with “degrees of health” 1 and 2 were considered Alive Algarrobo whereas “degrees of health” of 3 were considered Dead Algarrobo With regard to quantification, only trees were taken into consideration, Overo, as a bush, was identified but not quantified. Error matrix derived measurements along with detection rates were employed to assess the accuracy of the classification and the tree location respectively. Further assessments such as determining the quality of tree crown delineation (i.e. the boundary of the tree crowns) could be implemented but while the actual delimitation of the crown boundaries has a direct influence in the accuracy of both tree detection and species classification it is not a focus of this study.

To examine the species composition in the study area we summarised the numbers of trees per species, numbers of trees, crown cover and mortality rates of Algarrobo, across the study area, using 50 x 50m cell sizes (area of 2500 m^2^). This was processed in ArcGIS 10.4 (ESRI 2016), deriving the cells using fishnet and summary statistics using spatial overlay (centroids of the crowns queried against the cells) and zonal statistics.

## 3. Results

**Fig 6:**
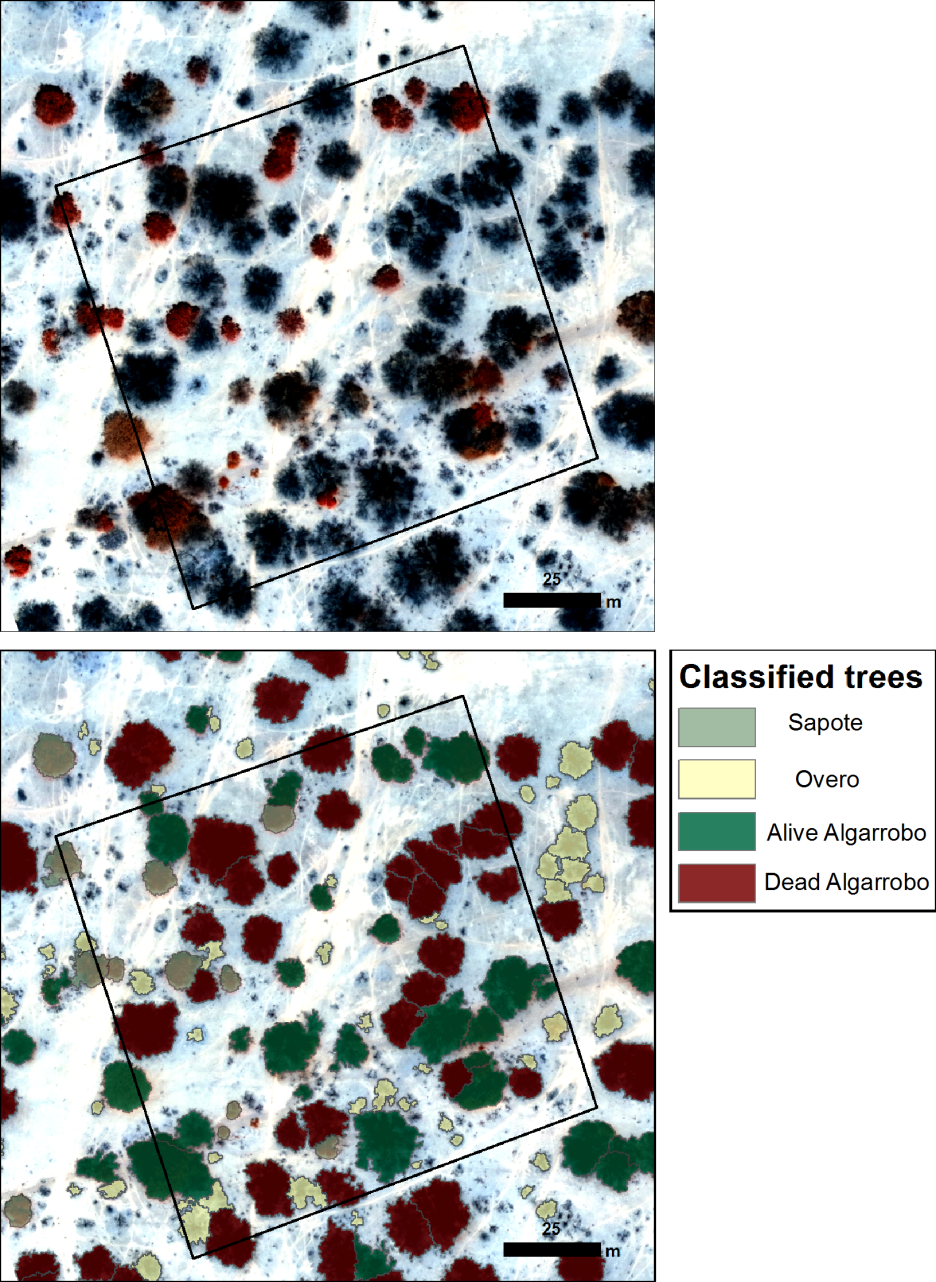
Distribution of species composition in and around Plot 1: A. red edge derive image from UAV mosaic. B Results from crown delineation with species identification.

**Fig 7:**
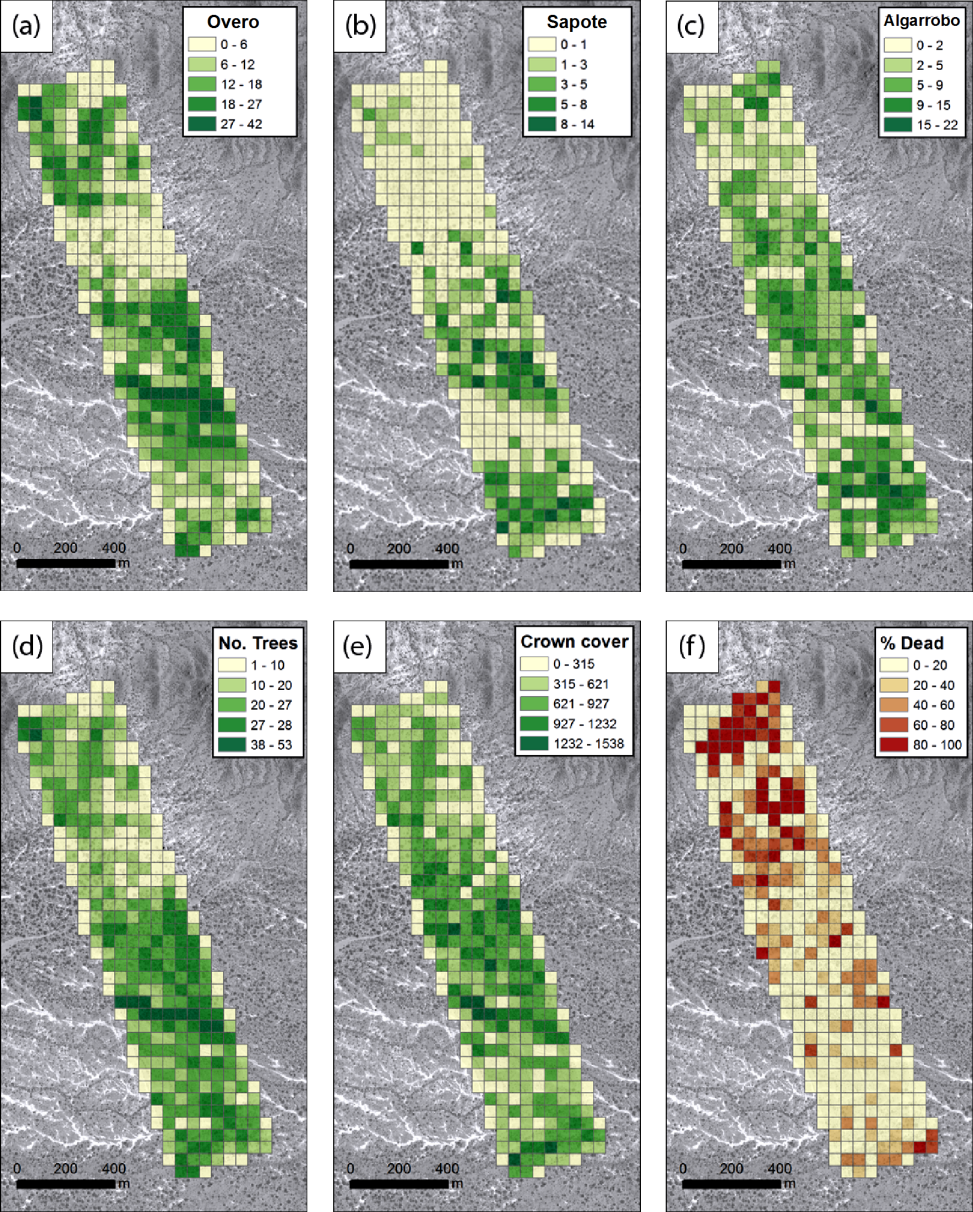
Landscape metrics for targeted species with 50 x 50 m cells (2500 m^2^). (a) Number of Overo per cell. (b) Number of Sapote per cell. (c) Number of c per cell. (d) Number of trees per cell. (e) Crown coverage in m^2^ per cell. (f) Percentage of dead Algarrobo per cell.

The distribution of species composition across the landscape (Fig 6) reveals the predominance of *Algarrobo* at 69% of delineated tree canopies (i.e., at 21.5 trees per Ha) as opposed to Sapote at 31% of the delineated trees (Overo was mapped but not quantified) (Table 2). At the time of the UAV deployment it is evident that 35% of the mapped *Algarrobo* trees were already dead (Table 2). When reviewing the spatial distribution of species (Fig 7), it is evident that *Overo*, is densest along runnels, whilst Sapote, dominates in the south of our study area. *Algarrobo* shows little trend, but what is evident is the clustering of the dead trees in the north of the area. There could be multiple reasons for this, such as the disease advancing from north to south but also the area to the north is the most disturbed (in terms of cattle and human disturbance) which may help propagate the plague.

**Table 2:** Number and density of trees in the study area.

	Number of trees	Adjusted number of trees	Number of trees per Ha	% Trees	% Dead
*Sapote*	743	784	9.8	31	
*Algarrobo* (alive)	1331	1122	14.0		
*Algarrobo* (dead)	504	601	7.5	69	35
**TOTAL**	**2578**	**2507**	**31.3**		

**Fig 8:**
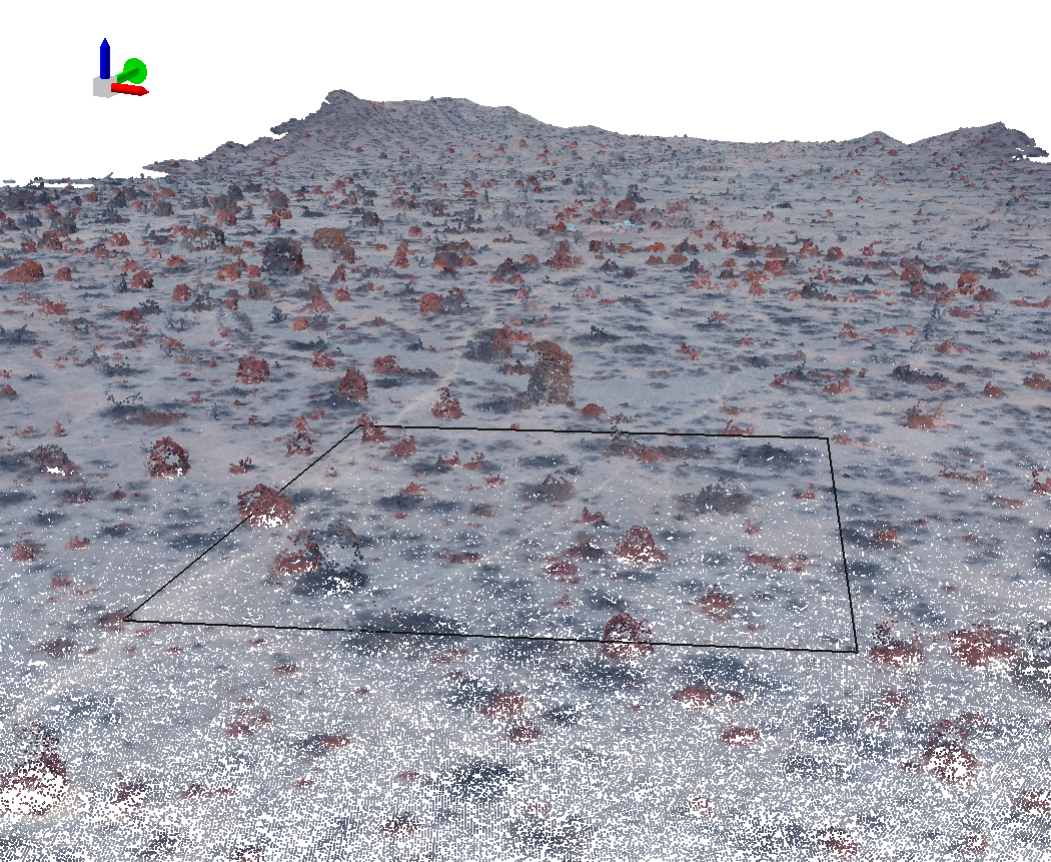
Point cloud showing higher density for evergreen trees as opposed to deciduous or defoliated tress.

The high degree of confidence in this mapping is supported by the accuracy assessment statistics produced. With respect to number of trees delineated, an overall detection rate of 95.3% was obtained (Table 3). When examining detection success per tree species it is evident that there is a general underestimation, except for the alive *Algarrobo* where there is an overestimation. This is most likely caused by distinct alive branches in diseased trees being recorded as a separate tree. Detection rates were used to adjust the final number of trees (by applying percentage of detected trees to the final map figures). With respect to accuracy of species identification of those delineated trees an overall accuracy of 94.10% was obtained (Tables 4 and 5). Optimal classification results are yielded by Sapote with a 100% user’s accuracy, although a lower producer’s accuracy (88.16%) was obtained, mainly due to alive *Algarrobo* trees being classified as Sapote. This is very much related to crown delineation errors; if the crown is delimited in such way that includes non-vegetation pixels (such as background) the average GDVI values of the tree object would be lower and therefore the tree assigned to a different class. The dead *Algarrobo* class presents a similar accuracy statistic behaviour (i.e., a high user’s accuracy and lower producer’s accuracy) and this is caused mainly by Dead *Algarrobo* trees being classified as *Overo*. This can be explained by inaccuracies in the elevation data; if not enough elevation points are found in the point cloud for the object tree, it will not be recognised as a tree but as a bush and therefore assigned to *Cordea lutea* class (Fig 8). In addition, some of the dead *Algarrobo* were identified as alive *Algarrobo* generally caused by *Vallesia sp*. plants growing under the *Algarrobo* canopy increasing GDVI values and resulting in the tree being classified as alive. This situation causes different accuracy statistics in the two remaining classes, Alive *Algarrobo* and *Overo*, where high producer’s accuracy than user’s accuracy was obtained. Most of the reference Alive *Algarrobo* were identified correctly whereas trees classified as *Algarrobo* were indeed Sapote.

**Table 3:** Accuracy assessment: Detection rates.

	Classified	Reference	Difference	%
*Sapote*	72	76	-4	94.73684
*Algarrobo (alive)*	59	51	8	115.6863
*Algarrobo (dead)*	73	87	-14	83.90805
Total	204	214	-10	**95.3271**

**Table 4:** Accuracy assessment: Error matrix.

		Reference Data				
		Capparis *scabrida*	Cordia *lutea*	Prosopis pallida (alive)	Prosopis pallida (dead)	Row Total
**Classified Data**	Capparis *scabrida*	67	0	0	0	67
	Cordia *lutea*	1	225	1	9	236
	Prosopis pallida (alive)	8	1	49	4	62
	Prosopis pallida (dead)	0	1	1	74	76
	Total	76	227	51	87	**441**

**Table 5:** Accuracy assessment.

	Reference Totals	Classified Totals	Number Correct	Producers Accuracy	Users Accuracy
Capparis *scabrida*	76	67	67	88.16%	100.00%
Cordia *lutea*	227	236	225	99.12%	95.34%
Prosopis pallida (alive)	51	62	49	96.08%	79.03%
Prosopis pallida (dead)	87	76	74	85.06%	97.37%
Totals	441	441	415		
Overall Classification Accuracy			94.10%		

## 4. Discussion and conclusions

The high degree of accuracy with which the target species were mapped across this landscape as well as mortality rates of the keystone species *Algarrobo*, demonstrates how useful a monitoring approach using a UAV would be for conservationists working in this area. Although much up front work is need to deploy the UAV, the actual flying time and image acquisition was very fast; covering the area in less than 20 mins.

Of particular note, is that although there are currently many studies reporting advances in the use of UAVs [37,59,60], this study has demonstrated that the simple and cheap consumer grade sensor carried by the system (Canon S110 RE), with its limited spectral resolution, still afforded accurate mapping of the parameters required here. However, when using modified consumer digital cameras as multispectral sensors we need to be aware of certain limitations of these systems to monitor vegetation. Spectral sensitivity and radiometric distortions of the camera optics have an influence in the radiometric and spectral quality of the images acquired [61]. Even if the camera is calibrated, attempting to remove radiometric distortions, theses cameras are sensitive to fairly broad wavelength ranges which also overlap considerably in detriment of traditional multispectral analysis. Furthermore the Red Edge is acquired with the loss of the red band which also limits the range of band indices that can be applied. Still, data derived from this modified cameras provide extremely valuable information for monitoring vegetation if the appropriate image analysis techniques are used. In addition to the ultra-high resolution data, the ability of the system to provide three dimensional information (derived using structure from motion) was useful for vegetation characterization but the contribution was highly dependent on the quality of the point clouds produced. Different studies report on accuracies comparable to those derived by LiDAR systems [62,63]. However, as others have found, there were challenges when generating digital canopy height models of deciduous trees [64]. It was evident that point clouds were dense for evergreen trees with compact canopies but less so for deciduous or defoliated tress where fewer tie points were found in standing branches which could be confused with background points (Fig 8). As a result 3D data was useful when delineating and discriminating evergreen species but not so conclusive for deciduous trees. However, the methodology approach used in this study allowed for the combined use of 3D and spectral information depending on the nature of the vegetation where spectral information was found more useful in dealing with deciduous trees. Nevertheless the use of 3D information is essential when trying to discriminate Dead *Algarrobo* tress from *Overo* bushes.

Fundamental to this study was the capture of ultra-high spatial resolution data, the ability to derive 3D data, as a result of structure from motion, and extending the spectral resolution of the camera sensor by way of a filter. Although UAVs are relatively easy to deploy, there is the caveat that understanding how they should be deployed with respect to flight planning to capture the optimal data for pre-processing and then subsequently the methods for processing and analysing the data captured for an application are key to successful mapping and monitoring. General pixel based quantitative analysis (relation between pixel values and target radiance, time series analysis, quantification of surface parameters, data comparison across time and scale) is less suitable than object based approaches where spatial and contextual information is also taken into account. The high resolution of the 3 band orthorectified data, such that the features to be mapped are larger than the pixel [65], and derived CHM were well suited to the employment of OBIA for mapping the species of individual trees. Previous studies delineating crowns have used a range of different methodologies, predominantly by applying edge detection [66], watershed segmentation [67] or region growing [68] algorithms, but these have been on remote sensing data captured by other platforms. These include multispectral very high resolution (VHR) satellite imagery [54], airborne LiDAR data [66] or a combination of both [69]. Such studies have normally focused on either temperate forests [54,70] or monospecific tropical environments [71] with relatively simple tree crown structures. Comparatively little research has tried to tackle the complexity of tropical forests with intrinsic interspecific low spectral separability and variable physical parameters [69]. Just a handful focus on these drier environments such as savannah woodlands [67], but so far these methodologies have not yet been applied to dry forests with the added complexity of trees presenting different levels of infestation by a fungal disease. Thus, this study is one of the first studies to demonstrate how OBIA can be successful in analysing data captured by UAVs, particularly the derived 3D models and multispectral imagery from uncalibrated consumer grade digital cameras. This study has also illustrated that the subsequent species classification process post-crown delineation has also benefited from the use of OBIA where information on the trees as objects including contextual information, statistical parameters based on spectral information (such as mean values, standard deviations…) provides an advantage over the use of traditional pixel based approaches when identifying individual species [72,73].

The results obtained here are extremely encouraging. They demonstrate that object-based image analysis is an effective image processing technique to analyse very high resolution data allowing for the identification of individual tree species and composition across a heterogeneous landscapes. It is also apparent that from an operational perspective, the relative ease of deploying the SenseFly eBee system across this landscape, in combination with the relative accessibility of using this type of UAV (i.e., costs, training, etc) means that repeat data capture (temporal resolution) should be achievable. Indeed, the need for rapid deployment and repeat data capture is particularly acute across this landscape as Algarrobo has throughout Peru been suffering dieback with the loss of foliage through the combined condition of drought, climate change and defoliating plagues. As a result the *Algarrobo* of our study area, are in a state of rapid decline and mortality, reaching alarming figures of a 35% mortality rate. There is an urgent need of action to reverse this trend putting in place community based conservation activities and restoration initiatives to ensure the survival of Algarrobo forests. Furthermore, extreme events (such as ENSO) are occurring more frequently [74] and their impact on the forest requires monitoring. However, to truly develop a UAV-based monitoring system that is flexible and allows for automation of workflow where possible spectral data derived from the UAV sensors should be calibrated ensuring long term data quality [75] as well as data comparability through time and space [76]. Additionally to fully exploit the multispectral capability of the system using consumer grade digital cameras, post-flight combination of data from different sensors should be considered allowing for a wider range of multispectral bands enhancing the analytical capability (such as vegetation indices) of the system and therefore being able to capture subtle differences in infestation levels. This would in turn raise the need of accurate ground control points and minimal time delay between flights [33].

The aforementioned improvements in the system are feasible however the present analysis has exposed the state of the vegetation and served as a baseline for future monitoring allowing to further focus on the health (i.e. levels of infestation) of *Algarrobo*. Moreover, once established such a UAV-based monitoring system it could be extended to compute extra individual tree information of great value for many ecological studies [77]. Measures of canopy extent (crown width, crown cover, foliage projected cover…) and tree heights values can be easily computed once the right crown delineation algorithm is in place [78], furthermore, tree height and crown width are known to correlate with other tree based metrics such as DBH [69,79]. These tree structural attributes are fundamental for assessing above grown biomass, carbon stocks and for the understanding of ecosystem functions which are essential in support of many conservation activities and the provision of ecosystem services [78]. To be able to do this, as well as identify further species would represent an exciting development for the fields of remote sensing, ecology and conservation [80]. With respect to this landscape in particular, it is clear that what is required of a monitoring system, i.e., provision of spatial data on the principal keystone species, including on their health, in a timely fashion on demand, should indeed be possible. The data generated via this study allows for the isolation of areas needing conservation management and planning, for example targeting areas of healthy trees for seed collecting, and conversely those areas needing assistance in restoration and intervention.

As for the generality of the methodology used, the forest of the study area is at the dry end of the spectrum for tropical dry forest where rainfall is very low resulting in a vegetation type with low number of species. Disturbances in the area have also caused an open structural vegetation formation where tree crowns are in many cases isolated and have developed freely to their mature form but also in tree clusters where crowns from individual trees are interconnected. The methodology used in this study, working at different levels (isolated trees and tree clusters) allows for this approach to be used in different structural vegetation types, either in closer formations such as forests receiving more rainfall where tree canopies are completely interconnected, or in very open formation such as savannahs (e.g. Brazilian cerrado). In more species rich vegetation types such as cerrado, the approach here would still be valid but would benefit from higher spectral resolution, either from an upgrade of UAV sensors or post-flight combination of data in order to fully exploit the multispectral capability for species identification.
